# Biostimulants Application: A Low Input Cropping Management Tool for Sustainable Farming of Vegetables

**DOI:** 10.3390/biom11050698

**Published:** 2021-05-07

**Authors:** Mohamad Hesam Shahrajabian, Christina Chaski, Nikolaos Polyzos, Spyridon A. Petropoulos

**Affiliations:** Department of Agriculture Crop Production and Rural Environment, University of Thessaly, 38446 Volos, Greece; cchaski@uth.gr (C.C.); npolyzos@uth.gr (N.P.)

**Keywords:** biostimulants, seaweed extracts, organic farming, arbuscular mycorrhizal fungi, protein hydrolysates, amino acids, chitosan, phosphite, plant growth promoting bacteria, humic substances

## Abstract

Biostimulants, are a diverse class of compounds including substances or microorganism which have positive impacts on plant growth, yield and chemical composition as well as boosting effects to biotic and abiotic stress tolerance. The major plant biostimulants are hydrolysates of plant or animal protein and other compounds that contain nitrogen, humic substances, extracts of seaweeds, biopolymers, compounds of microbial origin, phosphite, and silicon, among others. The mechanisms involved in the protective effects of biostimulants are varied depending on the compound and/or crop and mostly related with improved physiological processes and plant morphology aspects such as the enhanced root formation and elongation, increased nutrient uptake, improvement in seed germination rates and better crop establishment, increased cation exchange, decreased leaching, detoxification of heavy metals, mechanisms involved in stomatal conductance and plant transpiration or the stimulation of plant immune systems against stressors. The aim of this review was to provide an overview of the application of plant biostimulants on different crops within the framework of sustainable crop management, aiming to gather critical information regarding their positive effects on plant growth and yield, as well as on the quality of the final product. Moreover, the main limitations of such practice as well as the future prospects of biostimulants research will be presented.

## 1. Introduction

The growing need for food production through sustainable cultivation practices, without reducing crop yield and producer income, is a major objective due to increased environmental pollution and the gradual degradation of cultivated soils [1]. In the context of global climate change and food security, there is a need for cultivating crops under unfavorable conditions, particularly in dry and semi-dry areas, as well as for the sustainable use of valuable and finite natural resources through the protection of biodiversity [2,3,4]. Various farming systems have been suggested throughout the last decades with biostimulants being a novel and sustainable approach towards crop production, especially under biotic and abiotic stressors [4,5]. The expected market growth in the biostimulant sector at a compound annual growth rate of 11.24% and up to USD 4.9 billion by 2025 [6]. Therefore, there is increasing interest in the farming sector for new biostimulant products and a lot of research is carried out in this gradually evolving section of the industry. There are several commercial products available which are currently applied on various crops within the context of sustainable and organic farming [7].

**Figure 1:**
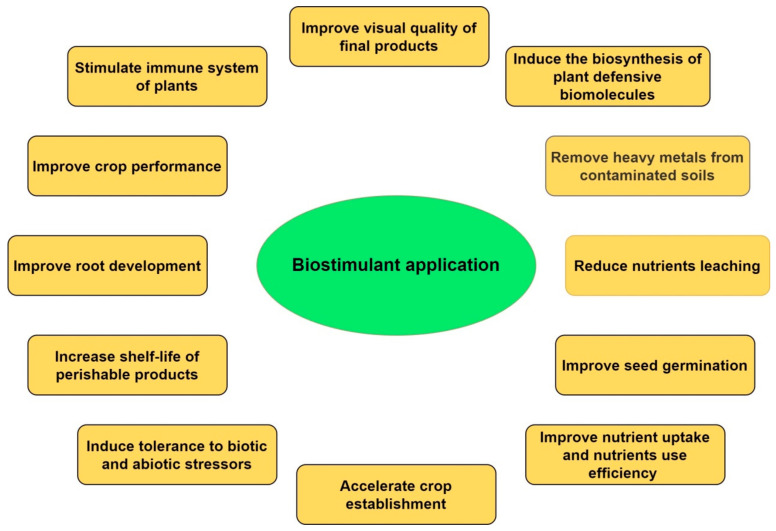
The most important biostimulant effects on crops.

Various compounds with bioactive properties can be utilized as biostimulants to boost plant growth and development under normal and stressful conditions [8,9], while among the distinctive characteristics a biostimulatory product must improve nutrients use efficiency, tolerance to abiotic stressors, quality of the final product and nutrients availability in soil [10]. So far, six distinct categories of biostimulants are recognized, including microbial inoculants, humic substances, such as humic and fulvic acids, protein hydrolysates and amino acids, biopolymers, inorganic compounds. and seaweed extracts, all of which are commercially available with wide applications in agriculture [11,12]. Biostimulant application can be considered as an effective and sustainable nutritional crop supplementation and may alleviate the environmental problems associated with excessive fertilization [5,13]. In intensive cropping sectors such as in horticulture and floriculture, the biostimulants can also increase nutrient use efficiency, partly substitute the chemical fertilizer inputs and ameliorate the yield and quality of crops [14,15,16]. However, biostimulants are not only considered as important substitutes to mineral fertilizers, but also notable in organic farming systems within sustainable crop production management [17]. Increased root and shoot growth, improved resistance against stressors, better root growth potential, and reduction in nitrogen fertilizer inputs are some of the most noteworthy impacts of biostimulant application in sustainable agriculture system [1]. Most of these impacts could be attributed to their auxin-like effect, as well as to the improvement in nitrogen uptake and metabolism, the regulation of K/Na ratio, and the proline accumulation which serves as an osmoprotectant against salinity stress [18,19,20]. Moreover, biostimulatory compounds may also have a positive impact on soil biology and they can be recognized as a good strategy for recovering semiarid areas and degraded ecosystems [21,22,23]. However, the variable composition of raw materials used for the production of biostimulant products make the task of revealing the mechanisms of action more difficult and long-term studies and standardization processes are needed [24]. The major biostimulant impacts on crops are shown in Figure 1.

Considering the numerous literature reports during the last decade related to biostimulants and their effects on various crops, this review aims to present the most up-to-date key results for biostimulant practical applications on crops and the new tools available for the unraveling of mechanisms behind the observed effects. In the present review, all relevant reports in English language were collected. The literature search was performed by using the keywords of plant biostimulants, seaweed extract, leafy vegetables, phenolic compounds, arbuscular mycorrhizal fungi, and biofertilizers in main indexing systems including PubMed/MEDLINE, Scopus, the search engine of Google Scholar, as well as the Institute for Scientific Information Web of Science.

## 2. Biostimulant Categories

Biostimulants are classified into two distinct classes based on their origin. Therefore, one category includes all those products that have biological origin being obtained from pathogens or from the plant itself and the second category includes all the products that do not have biological origin such as physical factors and chemicals [25]. Moreover, biotic biostimulants may have a defined composition and contain molecules of known structure or being more complex including several molecules with different structures [26]. Another classification approach divides biostimulant products in microbial, which are obtained from arbuscular mycorrhizal fungi and plant growth promoting bacteria, and non-microbial biostimulants which include plant micro-algae extracts, humic substances and biopolymers such as chitosan [27,28,29,30,31]. In particular, the microbial biostimulants may promote plant growth both directly and indirectly; biofertilization, stimulation of root growth, tolerance to plant stressors and rhizoremediation are a few examples of direct effects on plant growth promotion [28,31], while controlling plant pathogens and enhancing the enzymatic activity of plants may indirectly induce plant growth [29,30]. Finally, many researchers divide the non-microbial biostimulants in phytohormonal and non-phytohormonal (those biostimulants that include protein-containing compounds) [32].

**Figure 2:**
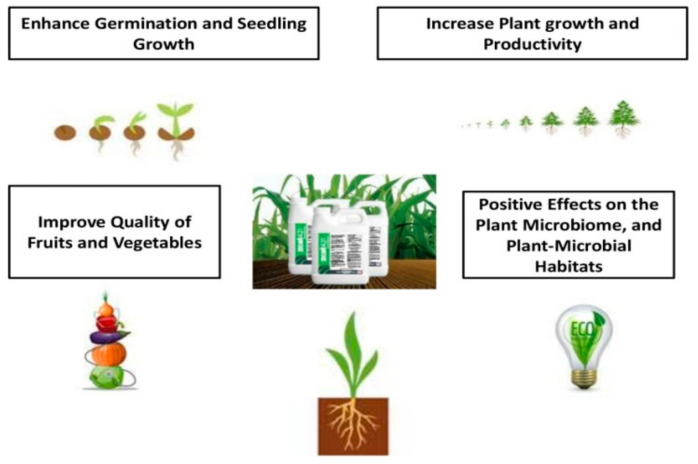
Protein hydrolysates biostimulatory effects.

Among the various compounds with biostimulatory activity, protein hydrolysates are in the spotlight of scientific research due to their promising properties. Such compounds are actually a mixture of amino acids and soluble peptides, which are mainly produced after enzymatic, thermal and chemical processes and derived from animal or plant origin proteins [33,34]. Their positive effects are associated with the up-regulation of metabolites involved in plant growth processes and the elicitation of hormone-like activities which altogether affect plant growth and productivity [20,35]. The most important benefits of protein hydrolysates are presented in Figure 2.

Similarly, seaweed extracts are also a widely known category of biostimulants with a steadily increasing penetration into the farming sector during the last decades. These compounds have found applications in various crops since they may induce tolerance against abiotic stressors and boost crop performance while they may also improve the shelf-life of various crop products [36]. They are largely prepared from brown seaweeds, such as *Ascophyllum nodosum*, *Ecklonia maxima*, and *Macrocystis pyrifera* and they consist of promoting hormones or trace elements such as Fe, Cu, Zn, and Mn [37,38]. Other compounds such as phloroglucinol and eckol are active biomolecules obtained from the brown seaweed *Ecklonia maxima*, which is one of the most common species of Kelp which is widely utilized as liquid fertilizer [39]. Moreover, the use of extracts from seaweeds that are industrially processed for other purposes (e.g., the production of carrageenan from *Kappaphycus alvarezii*) may reduce the carbon footprint of industrial sector and increase at the same time the added value of seaweeds [40].

*Trichoderma*–based biostimulants are another important category of microbial biostimulants that have found applications in crop production since they may improve plant nutrient status and tolerance against environmental stressors stress via the boost of root growth, the increased nutrient uptake and the production of auxins and secondary metabolites (e.g., peptides, volatile organic compounds) [41,42,43,44,45]. However, several other fungi have shown biostimulatory activity on crops with beneficial effects on plant growth and yield and response to oxidative stress [46].

On the other hand, the number of plant growth promoting bacteria (PGPB) used in various formulation is quite low when considering their great biodiversity [47]. PGPB and symbiotic microorganisms may act through various mechanisms related to hormone release or changes in hormonal balance within plants, the improvement in nutrients availability, the biosynthesis of volatile organic compounds, and the increased tolerance to abiotic stressors through the induction of systemic tolerance [17,48,49,50]. The main negative effects of abiotic stressors (e.g., salinity, drought) on plants are related to changes in endogenous hormones balance (e.g., ethylene production, increase of absicic acid and decrease of cytokinins levels) which results to reduced shoot and root growth as a means to plant homeostasis regulation [51]. This is the key point where PGPB come into play since they promote the production of indole acetic acid which in turn alters root architecture and induce root development resulting to a larger root area and to more root tips. In more detail, these additional (exogenous) phytormones along with the already existing hormones in plant tissues (endogenous) regulate cell proliferation (especially in the roots) which facilitate the uptake of water and minerals from required to support plant growth [52]. Roots and shoots may communicate through hormonal signaling and actually roots may regulate the development and growth of the aerial parts of the plant by transferring endogenous hormones via the xylem to the shoots which act as hormonal sinks [53]. However, apart from the endogenous hormones which plant may produce itself, several other phytormones have been detected in the root-soil environment related to the soil microbiome which may enter the plant through the transpiration flow (e.g., xylem) and regulate plant growth depending on their balance [54]. Soil detected phytormones may be produced from plant roots acting as signals for root functioning, or by soil microbiome (bacteria and fungi) [55]. The overall balance of these ex planta hormones is regulated by biosynthesis and uptake from roots, as well as by production, uptake and degradation of hormones from soil microbes [54], and interacts with in plant hormones, thus regulating plant growth and development [51]. Moreover, arbuscular mycorrhizal fungi (AMF) and rhizosphere microflora combinations seem to be effective not only in improving crop productivity but also in preserving soil health and fertility [56].

Humic-like substances such as humic and fulvic acids may also exhibit biostimulatory activity, since various reports have suggested improved crop performance attributed mostly to auxin- and cytokinin-like effects [57,58]. They are derived from organic matter decomposition and metabolic products of soil microbes and they contribute to plant growth through the improvement of soil physic-chemical properties and the increased availability of nutrients in rhizosphere [7]. The main effects of humic substances are in general the improvement in root growth and morphology, the increase in the uptake of nutrients and their use efficiency, the better crop performance, and finally the increase in fruit quality and in tolerance against abiotic stressors [59,60]. The actual mechanisms of action seem to be the result of synergistic between the various bioactive compounds that raw material include, although the effects may differ depending on the crop, the soil type and soil microbes present in the rhizosphere [61]. In addition, humic and fulvic acids may promote plant growth through hormone-like effects, since the breakdown of these substances releases auxins and other pre-cursors [58,62,63,64]. Moreover, Canellas et al. [65] suggested a hormone-like activity of humic substances fraction on tomato plants through the release of auxin-like biomolecules. Considering that humic-like substances and humic acids in particular can be obtained from various raw materials such as natural organic matter, plant tissues and bio-waste, they present a variable composition with heterogenous effects, depending on their molecular weight [65,66].

Phosphite (Phi) and biopolymers such as chitosan were also reported to possess biostimulant properties with several applications on horticultural crops [67,68]. Regarding Phi, it is widely used as fungicide against various pathogens or as a supplement of P nutrition in crops; however, its application is also associated with plant growth promoting effects which are attributed to promoted root growth and better uptake and assimilation of nutrients from plants [67,69]. On the other hand, chitosan is a biopolymer produced after the deacetylation of chitin and is in the research focus during the last decades due to the interesting effects on crops [68]. It is commercially produced from seafood shells and its main application is related to plant defense against pathogens, since it may induce the production of protective molecules against pathogens [70]. Biostimulant activities have also been reported being mainly associated with increased photosynthetic activity, tolerance to drought, salinity, and extreme temperatures stress and activity of antioxidant enzymes [71,72]. However, considering that chitosan is a biopolymer that comprises compounds of different deacetylation and polymerization degree there is a great variability in the composition of the commercially available products which may also result in variable effects on crops [73].

Apart from these well-established categories, there is significant interest from the biostimulant sector for waste and by-products which exhibit important biological activities and they could be considered as a new category among the existing ones creating alternative pathways in for by-products management [74,75]. In this context, the production of dissolved organic matter (DOM) through anaerobic digestion has shown promising results for the design of new biostimulatory products that may improve plant health through an auxin-like mode of action [66]. According to Messias et al. [76], shale water, which is generated after the pyrolysis of pyrobituminous shale rock, has also shown important biostimulant effects in horticultural crops and could be used as a yield enhancer and biofortifying agent. Finally other compounds such as melatonin and vitamins have shown biostimulatory activities, especially under abiotic stress conditions, and apart from the induction of secondary metabolites biosynthesis they also improve the quality and the functional properties of the final products [77,78,79].

Considering the novel status of the biostimulants sector, as well as the fact that various substances and organisms can be classified as biostimulants by definition, biosafety criteria are important for choosing new microorganisms as biostimulants, and biosafety measures should be addressed according to bioassays rather than on taxonomy and based on environmental and human safety indices (EHSI; [80]) [81]. Moreover, any negative effects associated with unintended effects on reactive nitrogen losses need more attention [82]. The main research topics for the biostimulant characterization of new substances and compounds characterization include, (1) evaluation of the biostimulant composition; (2) standardization of the production methods; (3) characterization of plant responses especially in combination with environmental conditions; (4) identification of crop-specific responses to biostimulants products; and (5) fine-tuning of application timing and doses [83]. The principal classification of plant biostimulants are shown in Table 1.

## 3. Practical Applications of Biostimulants and Biostimulatory Products on Horticultural Crops

Various biostimulant products have been studied in numerous research reports. *Ascophyllum nodosum* extracts are among the most commonly studied biostimulants with varied effects on several crops such as the yield and nutritional quality of spinach [16,103,104,105], the nutritional status and shelf-life of lettuce [106], increased the drought tolerance in tomato plants [107], improved plant growth and yield in carrot and strawberry [108,109,110,111], or alleviated the water stress effects on common bean [15,112]. The mechanisms behind these beneficial effects of *A. nodosum* extracts are still under investigation, although various studies postulated hormonal effects on plant growth through the up- or down-regulation of auxin-responsive genes [113]. This argument is supported by the composition of *A. nodosum* extracts which contain several hormones (e.g., abscisic acid, auxins, brassinosteroids, cytokinins, ethylene, gibberellins, and strigolactones) [113], although the opposition suggests that the low hormones content along with the low application doses of biostimulants cannot justify these positive effects on plant growth [90,114]. However, the recent study of Dookie et al. [115] came to confirm the hormonal effects of seaweed extracts, since the foliar application of *A. nodosum* and *Sargassum* sp. extracts on tomato plants up-regulated the expression of six flowering genes. Other recent studies highlight the protective effects of seaweed extracts against oxidative stress in plants subjected to environmental stress, thus reducing electrolyte leakage and lipid peroxidation [116].

*Ecklonia maxima* is another brown microalgae the extracts of which have found several application in crop production via various biostimulatory products. The effects of these extracts have shown positive results on crop yield and leaf color of lettuce plants [117], on mung bean germination and plant growth [118], and on growth and nutritional quality of spinach [119]. The application of *E. maxima* extracts on potato plants also had varied effects including the increase of marketable yield [120] the improved tolerance to abiotic stress and total assimilation area [121] whereas contrasting effects on quality were reported with no effects on dry matter, protein, total sugars and vitamin C content [122] or increasing trends on total and true proteins content being observed [123]. The detailed analysis of the extracts identified new plant growth biostimulants, namely eckol and phloroglucinol [119,124,125], while other plant growth regulators such as abscicic acid, gibberellins and brassinosteroids were also detected in commercial products indicating a hormone-like activity [126]. Apart from these two algae species, several other macro- and microalgae extracts have been incorporated in commercial formulation that are currently used in various horticultural crops [127]. However, despite the scientific evidence regarding the hormonal effects of seaweed extracts, several factors associated with the variability in experimental set-ups, the plethora of seaweed-based products and their species-specific effects, the lack of information regarding the analytical composition of such products and the variable composition of raw material throughout the year make the definition of mechanisms of action difficult [113].

Another category of biostimulants widely used in horticulture is protein hydrolysates and nitrogen-containing compounds. There are several commercial products available derived from plant or animal proteins with various applications in horticultural crops during the last few years [128,129,130]. For example, one of the first studies was conducted on common bean with protein hydrolysates derived from tomato plant residues and reported a significant increase in nitrogen assimilation of bean [131]. Other crop residues were also promising sources of protein hydrolysates and have found practical applications in vegetable crops, e.g., tomato grown in organic [132,133] and conventional farming systems [134], or common bean plants grown under water-stress conditions [135]. Other application of protein hydrolysates refer to plants subjected to stress conditions such as in the study of Koleška et al. [136] who tested the effectiveness of biostimulants in alleviating macronutrient deficiency effects on tomato plants, or Casadesús et al. [137] and Ertani et al. [138] who studied the hormonal effects of plant biostimulants on water-stressed tomato plants. It is suggested that foliar or root applications of protein hydrolysates may improve root development, C and N assimilation and nutrients uptake from plants [33,139,140], regulate the metabolic processes through a multi-level signaling that involves auxin-like activities [141,142,143], as well as to increase the effectiveness of plant defense mechanisms against abiotic stressors in a sustainable manner [144]. However, apart from increased tolerance to stressors the application of protein hydrolysates may enhance quality parameters in fruit and leafy vegetables [35,105,145].

On the other hand, nitrogen containing products have also shown promising results in alleviating stress effects on horticultural crops, as in the case of spinach [16] and common bean [15]. Gelatin is another compound included in animal-derived protein hydrolysates which may improve vegetable crops performance through the up-regulation of nitrogen assimilation by plants [146]. The application of amino acids has also shown positive effects on plant growth, photosynthetic processes and nutritional quality of lettuce [147,148], nutritional quality and physiological parameters of common bean [149,150], plant growth and nutritional status of fennel [151], physiological parameters and chlorophyll content of broccoli [152], and plant growth and fruit quality of tomato grown under iron deficiency [19] or macronutrients deprivation regimes [153].

Humic substances (HS) include humic and fulvic acids which are present in soil organic matter as well in aquatic environments and the atmosphere and differ with each other in their molecular weight [88]. Their application in pepper plants resulted to an increase in plant growth and to accelerated fruit development without significant difference in terms of fruit yield being observed from the untreated plants [154]. In tomato plants, the exogenous application of HS in combination with chelated FeEDDHA increased iron uptake, while it also improved phosphorus content in leaves [155]. Moreover, HS application increased early yield in potato crop when plants were grown under low temperatures and water availability [156], while the incorporation of HS in growing medium increased seed germination and seedling growth in tomato and okra [157,158]. Similar positive effects of HS application were observed on garlic through the stimulation of N and S uptake [159,160], or on onion plants without however significant correlations between the yield and nutrients uptake [161]. The increased yield and quality of potato tubers after the incorporation of HS in soil was attributed to the better availability use efficiency of nutrients due to reduced leaching, as well as to increased water holding capacity of soil [162,163]. Humic substances may also alleviate negative effects of high salinity, as reported by Shalaby and El-Messairy [164] in melon plants. In contrast, Ibrahim and Ramadan [165] reported inconsistent results for the foliar application of zinc combined with humic acid and chitosan on common bean plants, while Hartz and Bottoms [166] suggested no significant effect of HS application on dry matter accumulation or fruit yield in lettuce and tomato, respectively. However, these contradicting reports could be associated with application time and doses, as already suggested by Bettoni et al. [167] for onion crop, or the application methods in the case of mung bean [168]. According to De Hita et al. [60], root application showed more consistent effects than foliar application of HS, since although hormone-like activities in both methods of application the foliar spraying has transient impact and has to be repeated during the growing period.

Biostimulants based on plant growth promoting microorganisms include microbial inocula from bacteria and fungi of various genera and have also found practical applications in horticultural crops, either alone or in combination with each other [169]. In the case of fungi, *Trichoderma*-based products are the most widely used in horticultural crops [170,171,172,173], although other fungi such as *Glomus* sp. are also applied in sustainable horticulture [15,27,41,174,175,176]. Regarding the plant growth promoting rhizobacteria (PGPR), the strategy to choose the appropriate ones includes six steps: (i) Determination of the target crop and commercial strategy; (ii) selection of growth media for the isolation of microbial candidates; (iii) screening for traits giving considerable agronomical advantages; (iv) screening for traits belonged to product development; (v) characterization of the mode of action of PGPR; and (vi) evaluation of plant growth efficacy [177]. However, considerable variability is observed in the obtained results and considering that mechanisms of action are not fully understood and the importance of soil physicochemical parameters it is advisable to analyze soil characteristics and then apply those PGPRs that best suit the conditions [48]. The application of PGPR is associated mostly with stress alleviation effects in various crops such as common bean, potato, and lettuce which consequently results to better plant growth and yield [48,178,179,180]. The plant growth promoting effects are related to hormonal regulation in plants, since soil microbes produce various phytohormones which enter the plant from roots via the transpiration flow and reach the shoot sinks where they can induce alterations in shoot and leaf morphology and physiology [181].

Phosphite is a novel biostimulant which may function as a phosphate source affecting plant growth and performance, as well as a biocide against various pathogens and abiotic stress reliever [67,97,182]. It is usually applied with foliar spraying or through the nutrient solution with drip irrigation in the form of potassium phosphite or phosphorus acid, resulting to beneficial effects on plant growth and yield in several vegetable crops [67,69]. The most profound effects were observed in potato crop, where the foliar spraying or potato seed treatment with potassium Phi-improved plant growth and yield and earliness of tuber maturity through the induction of defense mechanisms and the increase of mycorrhizal colonization [94,96,97]. However, there are also studies where negative effects were reported for Phi application on tomato and pepper due to phytotoxicity effects, especially when plants are subjected to P-deficient conditions [182]. According to the same study, the positive or negative effects of Phi are highly associated with the P availability of plants, since Phi per se is not an effective form for P supplementation of plants and positive effects are due to pathogens control [182].

Biopolymers, such as chitosan, have been widely used in horticultural crops cultivation for many years mostly for pathogen control purposes [85]. The modes of application include foliar spraying, direct incorporation in soil or coating of vegetable products [68,71]. In particular, chitosan application was found beneficial for lettuce and tomato plants growth [71,183] and increased phytochemicals defensive metabolites content in spinach leaves [72]. However, there is evidence that bulk chitosan is associated with root growth inhibition when applied in non-optimal concentrations, therefore, alternative forms have been suggested including chitosan micro- and nanoparticles which are safer for agricultural use [184,185].

Silicon (Si) has many biostimulant activities such as enhancing growth and development of horticultural crops, especially under abiotic stress conditions; Si mechanisms of action are involved in oxidative damage, water relations, photosynthesis, ion uptake, hormones, and acts mostly via silica deposition in tissues providing mechanical strength [10]. Although Si effects are mostly visible under stressful conditions, its application may also have beneficial effects on crops grown under optimal conditions since it improves photosynthetic activity and plant growth [186]. The main application form is the foliar spraying, soil incorporation or fertigation of silicic acid and silicates [187], although new forms have also been suggested, such as silicon nanoparticles, for better uptake of Si from plants compared to the bulk form [188]. There are several examples of beneficial effects of Si on vegetable crops, such as tomato [189,190,191], cucumber [192], pepper [193], and squash [194], where Si application alleviated the negative effects of abiotic stressors on plant growth.

A great variety of biostimulant products with different active compounds have been suggested for application in the agricultural sector, including phenolic acids [195], triglycerides [196], titanium [2,197], or zeatin from *Moringa oleifera* leaves [198]. Moreover, chitosan is another important biopolymer with biostimulant activities which has been used to alleviate water stress negative effects and increase shelf life on horticultural crops such as basil [92], lettuce [71], spinach [72], tomato [183], and pepper [184], among others.

Apart from single product effects, there are several reports where the combination of biostimulants resulted in beneficial effects on horticultural crops which usually are better than those of single biostimulants [145]. For example, microalgae combined with humic acids improved the growth and yield of onion [161], plant growth promoting bacteria acted synergistically with humic acids to improve the growth of tomato [199,200] and potato plants [201], *A. nodosum* extracts combined with humic acids enhanced plant growth and shelf life of lettuce and spinach [202], or the interaction of plant growth promoting rhizhobacteria with seaweed extracts from *E. maxima* which increased plant growth and photosynthetic pigments content in *Amaranthus hybridus* plants [203]. In this context, Rouphael and Colla [204] suggested complex synergistic and additive effects of microbial and non-microbial biostimulants with mechanisms of action that have to be unraveled at a molecular level aiming to design the new generation of biostimulant products.

The main effects of biostimulants on vegetable crops are summarized in Table 2.

## 4. Future Remarks and Conclusions

Sustainable farming of vegetables is the focal point of research within the last decade considering the ongoing climate change and the increasing incidences of weather extremities as well as the pressure on crops from other abiotic and biotic stressors e.g., water and salinity stress or pathogens infestations. Moreover, food security and the efficient use of natural resources are in a tug-of-war with increasing food demands on the one side and replenishment of natural resources and their efficient use on the other side. Conventional cultivation of vegetables under these conditions becomes more and more difficult and farmers throughout the world start to adapt sustainable cultivation practices seeking always new methods. Biostimulants application is proven a useful tool towards this aim, allowing vegetable producers to cultivate under unfavorable conditions without adverse effects on crop yield. Moreover, the great variety of biostimulant products means there are commercially available products suitable for various conditions and crops. The present review gathered the most up-to-date information regarding the classification of biostimulatory agents and their main mechanisms of action, as well as their practical applications on vegetable crops. Although there are several cases where biostimulant application resulted to beneficial effects on plant growth and yield, more studies are needed to fine-tune application practices, since it seems there are product and crop specificities to be addressed and negative or no effects are also reported. These variable effects reported in the literature are usually due to the variable composition of biostimulants which are natural matrices that include various compounds from different classes and different activities, as well as to uncertainties in application times, methods and doses. Finally, the crop factor is also important since the genotype has a great effect on the response to biostimulant products, especially under stressful conditions.

Considering the above, the production and application of is an evolving process and new biostimulant products are needed. However, this should be realized under a new approach focusing on the synergistic effects of various biostimulatory agents instead of single-product application. Moreover, studying the molecular mechanisms behind the observed activities will help to reveal those physiological and plant metabolism pathways involved in this process and provide farmers with tailor-made products suitable for variable conditions. The application of biostimulants is not just a promising and environmentally-friendly practice, but it may also lead to increased use efficiency of natural resources through water deficit irrigation regimes and the reduced input of agrochemicals (e.g., mineral fertilizers and chemical for pests and pathogen control). It can also increase the sustainability of agricultural and horticultural production systems as well as improve the quality and quantity of food for the ever-growing world’s population.

## Figures and Tables

**Table 1:** Classification of plant biostimulants.

Plant Biostimulants	Key Points	References
Protein hydrolysates (PHs) and other *N*-containing compounds (amino acids)	a. Mixtures of peptides and amino acids which are produced via enzymatic, chemical or thermal hydrolysis of animal- or plant-derived proteins.	[34,84]
	b. Effective in increasing yield and quality of various crop products.	[85]
	c. Categorization based on proteins^,^ sources and the hydrolysis system; PHs boost both primary and secondary plant metabolism biochemical and physiological procedures.	[86,87]
	d. Effective in alleviating negative abiotic stress effects.	[24]
Humic substances	a. Include fulvic acids and humic acids which they differ in color, molecular weight, carbon content and the degree of polymerization.	[88]
	b. They could increase cationic exchange capacity (CEC) of the soil and interact with root membrane transporters.	[65]
Seaweed extracts	a. Extracts from brown seaweeds, e.g., *Ascophyllum*, *Fucus*, and *Laminaria* genera.	[89]
	b. They are rich in polysaccharides, polyphenols and compounds with hormonal activity that affect plant growth and development.	[90,91]
Biopolymers (Chitosans and other polymers)	a. Chitosans are naturally occurring components in fungi nematodes, insects and crustaceans.	[68]
	b. They regulate plant-defense mechanisms related to phytoalexins biosynthesis, reactive oxygen species, and pathogenesis-related proteins making plants more resistant to biotic and abiotic stressors.	[92]
Microbial biostimulants (Mycorrhizal and non-mycorrhizal fungi, *Rhizobium*, *Trichoderma*, and Plant Growth-Promoting Rhizobacteria (PGPR))	a. Symbiotic fungi, especially arbuscular mycorrhizal fungi (AMF) within the genus *Glomus*.	[14,16]
	b. *Trichoderma* genus	[44]
	c. Beneficial bacteria with plant growth promoting properties also known as PGPBs (*Bacillus*, *Rhizobium*, *Pseudomonas*, *Azospirillum*, *Azotobacter*, and many others).	[48]
Phosphite (Phi)	a. A phosphate (H_2_PO_4_^−^) analog which affects various plant growth and development processes.	[93]
	b. Several beneficial effects have been reported in various vegetable crops.	[69,94,95,96,97]
	c. Biostimulatory impacts on fruit such as avocado, banana, citrus, peach, raspberry and strawberry.	[69,98,99,100]
Silicon	a. Effective against abiotic and biotic stressors.	[11]
Vermicomposts	a. Hormonal activity of vermicompost leachates due to content in trace elements of hormones such as cytokinins, indolo-acetic acid (IAA), eighteen gibberellins (GAs) and brasinosteroids.	[101]
	b. Phytohormones from three different classes, including cytokinins, auxins and gibberellins provide plant growth promoting activities in vermicompost	[102]

**Table 2:** Selected biostimulants effects on various vegetable crops.

Plant	Common Name	Key Points	Effects	References
*Allium cepa* L.	Onion	a. Biostimulants containing humic acids, organic substances, amino acids, carbon and boron or algae extracts	Improved plant growth and yield, and shelf life of bulbs	[205]
		b. Application of diluted bee-honey extract (DHE)	Increased photosynthetic parameters, biomass production and yield, and antioxidants activity	[206]
*Allium cepa* var. *aggregatum* L.	Shallot	a. Application of seaweed extracts, vermicompost and mixture of animal waste	Improved yield and bulb traits	[207]
		b. Soaking of seeds in PGPB biostimulants	Increased germination percentage, plant growth and bulb parameters	[208]
*Allium sativum* L.	Garlic	a. Foliar application of liquid humic substances obtained from vermicompost extracts	Improved yield and quality parameters of bulbs	[160]
*Amaranthus hybridus* L.	Amaranth	a. Foliar application of vermicompost leachate, smoke-water, karrikinolide, eckol and Kelpak	Increased growth, higher chlorophylls, carotenoids and proteins content	[209]
		b. Combination of plant growth-promoting rhizobacteria (PGPRs), and *Ecklonia maxima* extracts	Improved plant growth and photosynthetic pigment content, stress relief	[203]
*Brassica juncea* L.	Mustard green	a. Foliar application of vermicompost leachate, smoke-water, indole-3-butyric acid and *Ecklonia maxima* extracts on seedlings grown in soils from goldmines	Increased phytoremediative activities though the accumulation of heavy metals	[210]
*Brassica oleraceae* L.	Broccoli	a. Combination of foliar spraying with *Ascophyllum nodosum* extracts and watering with amino acids on broccoli plants subjected to water stress and re-watering	Increased photosynthetic parameters under water stress conditions	[152]
		b. Combination of foliar spraying with *Ascophyllum nodosum* extracts and watering with amino acids on broccoli plants	Increased total phenolic compounds, sinapic acid and quercetin content	[211]
*Brassica oleraceae* L.	Cabbage	a. Foliar application of eckol from *Ecklonia maxima* extracts	Increased root and shoot length, photosynthetic pigments and proteins, proline and iridoid glycosides; inhibition of infestation from aphids	[39]
		b. Thiosulfate application through the nutrient solution in cabbage plants subjected to Cd toxicity	Improved phytoremediative properties of Cd without biostimulant effects on cabbage plants	[212]
*Capparis spinosa* L.	Caper	a. Incorporation of crushed maize seeds in growing medium of caper plants subjected to salinity stress	Increased activity of soil enzymes, Na exclusion from plant tissues	[213]
*Capsicum annuum* L.	Pepper	a. Application of a lipo-complex biostimulant containing mainly polysaccharides, polypeptides and vitamins	Increased phenylalanine and metabolites associated with fruit ripening (organic acids, monosaccharides, carotenoids)	[214]
*Capsicum frutescens* L.	Chilli pepper	a. Foliar application of oligochitosan	Increased plant growth, chlorophyll content and fruit weight	[215]
*Coriandrum sativum* L.	Coriander	a. Seed inoculation with *Azotobacter chroococcum* and *Azospirillum lipoferum*	Increased biomass production	[216]
		b. Foliar spraying with biostimulants (Asahi SL or Goemar Goteo) on plants subjected to chilling stress	Increased photosynthetic parameters, L-ascorbic acid and total phenolic compounds content and total antioxidant activity	[217]
*Cynara scolymus* L.	Globe artichoke	a. Application of *A. nodosum* extracts and trace elements.	Increased number and weight of heads	[218]
*Daucus carota* subsp. *sativus*	Carrot	a. Foliar application of Kelpak SL and Asahi SL	Kelpak SL improved nutritional value and increased storage life of carrots	[219]
*Lactuca sativa* L.	Lettuce	a. Foliar and root application of protein hydrolysates in lettuce plants grown under salinity conditions	Mitigation of oxidative stress, increased osmolytes and glucosinolates content	[220]
		b. Foliar application of liquid humic substances obtained from vermicompost	Improved earliness of plants, increased the number of leaves per plant and total yield	[221]
		c. Application of crude seaweed extracts (*Gracilaria caudate* and *Gracilaria domingensis*) on lettuce seedlings	Increased root growth	[222]
		d. Inoculation of growth substrate with *Bacillus* spp.	Positive effects on plant growth nitrate content	[223]
*Manihot esculenta* Crantz	Cassava	a. Foliar application of *Moringa oleifera* leaves extracts. The plant height and leaf number of cassava plant were increased because of foliar application of MLE.	Improved plant growth and decreased incidence of *Zonocerus variegatus* attacks	[219]
*Nasturtium officinale*	Watercress	a. Foliar spraying of algal biostimulant on watercress plants grown in Cd contaminated soil	Increased plant growth and reduced Cd accumulation in plant tissues	[208]
*Ocimum basilicum* L.	Basil	a. Foliar application of *Moringa oleifera* leaves extracts	Increased growth and yield, and estragole and eucalyptol contents	[224]
		b. Foliar spraying with palm pollen grains extract alleviated the negative effects of deficit irrigation on basil plants.	Improved plant growth and essential oils content and antioxidant enzyme activities; maintained relative water content, electrolyte leakage and water use efficiency; improved leaf and stem anatomy	[225]
*Phaseolus vulgaris* L.	Common bean	a. Foliar application of protein hydrolysates from pumkin seeds on *Phaseolus vulgaris* plants grown under saline conditions	Maintained plant growth, yield and anatomical features; mitigated negative effects of salt stress on macronutrients, photosynthetic pigments, relative water content and stability of cell membranes	[135]
		b. Foliar and soil application of Nomoren, EKOprop, Veramin Ca on *Phaseouls vulgaris* plants grown under normal irrigation and water stress conditions	Positive effects on yield, nutritional parameters, chemical composition and bioactive properties of fresh pods and seeds	[14,15]
		c. Seed soaking and foliar spraying with licorice root extract on common bean plants subjected to salinity stress	Improved growth, yield and physicochemical parameters	[226]
		d. Seed soaking and foliar spraying with salicylic acid and *Moringa oleifera* leaves extracts on common bean plants subjected to salinity stress	Improved growth, yield and physicochemical parameters	[227]
*Phaseolus vulgaris* L.	Snap bean	a. Foliar spraying with *Moringa oleifera* leaves extracts	Improved plant growth and yield components, increased total phenolic compounds and minerals content in pods	[228]
		b. Foliar spraying with garlic cloves extracts	Improved plant growth parameters, yield and chemical composition of pods	[229]
*Pisum sativum* L.	Pea	a. Foliar spraying with *Moringa oleifera* leaves extracts	Increased biomass production, pod and seed yield, proteins and nutrients content in seeds	[230]
		b. Seed soaking in licorice root extract	Increased seedling growth, photosynthetic activity and antioxidant enzymes activity	[231]
*Solanum lycopersicum* L.	Tomato	a. Incorporation of humic acids and/or crushed maize grain	Improved shoot and root growth, increased relative water content and membrane stability of transplants, improved macronutrients uptake	[232]
		b. Seed pretreatment with liquid extracts of *Chaetomorpha antennina* green seaweed	Increased germination percentage and vegetative growth, improved biochemical profile	[233]
		c. Foliar spraying with Chitosan microparticles	Improved seed germination and seedling vigor, modulation of antioxidant enzymes activities	[234]
		d. Foliar treatment with saffron extract	Improvement in morphological and biochemical parameters, antifungal effects against *Phytophthora infestans*	[234]
		e. Foliar application of humic (Megafol) and amino acids (Viva) biostimulants	Improved plant growth under normal fertilization rates and minimized yield loses under nutrients deprivation	[153]
		f. Foliar application of Tecamin Brix^®^ and/or Tecamin Flower ^®^ in tomato plants grown in saline conditions.	Improved yield and fruit quality	[235]
		g. Deed treatment and foliar spraying of microalgal extracts	Improved germination and seedling growth rates	[236]
		h. Soil application of compost and arbuscular mycorrhizal fungi	Improved plant growth and photosynthetic parameters, reduced incidence of *Verticillium dahliae* infestations	[237]
		i. Soil application of biostimulants containing plant extracts, *Ascophyllum nodosum* extracts or animal derived protein hydrolysates in tomato plants after transplantation	Reduced transplantation shock through the increase of root and shoot development	[238]
		j. Fertigation with microalgae polysaccharides	Improved vegetative growth, increased nutrients, protein and sterols content in leaves	[239]
		k. Foliar application of brown seaweed extracts from *A. nodosum* and *Sargassum* sp.	Induced flower formation and fruit setting	[115]
*Solanum melongena* L.	Eggplant	a. Foliar application of *A. nodosum* extracts	Improved flower and fruit set, fruit yield and chemical composition	[240,241]
		b. Foliar application of aqueous garlic bulb	Single application increased plant growth, photosynthetic parameters and antioxidant enzymes activity	[242]
*Solanum tuberosum* L.	Potato	a. Combined application of Ecklonia maxima extracts and Asahi SL with herbicides	Increased content of true and total proteins, increased marketable yield and yield parameters	[120,123]
		b. Soil spraying of biostimulant containing *N*-fixing microbes (NFM0 combined or not with an amino acid blend	Unintended impacts on nitrogen losses	[82]
		c. Potato seed pretreatment and foliage spraying with phosphite	Induced structural and biochemical changes in tuber periderm and cortex, increased tolerance to UV-B, enhanced sprouts emergence and early growth	[94,95,97]
		d. Foliar application of biostimulants containing *A. nodosum* extracts, *E. maxima* extracts or humic and fulvic acids	Increased yield under drought stress, increased marketable yield	[156]
*Spinacia oleracea* L.	Spinach	a. Foliar spraying of smoke-water and *Ecklonia maxima* extracts,	Increased growth and biochemical parameters (antioxidant enzymes activity and sinapic acid content)	[119]
		b. Application of various biostimulants (Megafol, Aminovert, Veramin Ca, Twin Antistress and irrigation treatments on spinach plants grown under normal and water stress conditions	Improved nutritional value and bioactive properties	[16]
*Vicia faba* L.	Broad bean	a. Foliar spraying with *Bacillus licheniformis*, yeast (5 g/L), extracts form algae and humic acid (20 g/L), increased pigments, carotenoids concentrations and total carbohydrates.	Improved photosynthesis and nutrients uptake, induced endogenous hormones and protein biosynthesis	[243]

## Data Availability

Not applicable.
